# The Health and Economic Benefits of United States Investments in Measles and Rubella Control and Elimination

**DOI:** 10.3390/vaccines12111210

**Published:** 2024-10-25

**Authors:** Kimberly M. Thompson

**Affiliations:** Kid Risk, Inc., Orlando, FL 32819, USA; kimt@kidrisk.org

**Keywords:** measles, rubella, vaccine, United States, health economic, modeling

## Abstract

**Background:** Prior to measles vaccine introduction in 1963, measles virus caused hundreds of thousands of annual reported cases, which led to substantial US morbidity, mortality, and costs. Similarly, congenital rubella syndrome (CRS) led to highly visible and tragic lifelong disability for thousands of Americans, before rubella vaccine introduction in 1969. The US certified national virus transmission elimination of indigenous measles in 2000 and rubella in 2004. **Methods:** Applying an existing integrated transmission and economic model, this analysis characterizes the net benefits of US investments in measles (1963–2030) and rubella (1969–2030) immunization assuming continued high routine immunization coverage. Due to importation risks, the US maintains two doses of both vaccines in its routine immunization schedule. **Results:** This analysis estimates total US costs of 8.1 billion (economics reported in 2023 US dollars) for measles immunization for 1963–2023 and 14.1 billion for rubella immunization for 1969–2023. The analysis estimates an additional approximately 1.2 billion for measles immunization and 1.5 billion for rubella immunization expected for 2024–2030. Historical and future US investments prevented an estimated approximately 237 million measles infections, 228,000 measles deaths, 193 million rubella infections, and 166,000 CRS cases. These investments imply net benefits (from avoided treatment costs minus immunization costs) of approximately 310 billion for measles and 430 billion for rubella and CRS, even without incorporating avoided productivity losses and intangible costs. **Conclusions:** US investments in measles and rubella immunization continue to provide enormous savings of human and financial costs and to prevent substantial mortality and morbidity.

## 1. Introduction

Aggressive control and elimination measures successfully ended the transmission of indigenous measles and rubella viruses in the US, but the importation of both viruses remains a concern for public health authorities despite over 55 years of the use of measles and rubella vaccines. Prior to vaccine introduction, the US annually incurred significant morbidity, mortality, and costs associated with the reported hundreds of thousands of measles cases and tens of thousands of rubella cases, and many more cases most likely went unreported. Measles infections cause rash and fever, and can lead to serious complications, including encephalitis, blindness, and death [1]. Infections with rubella virus (also known as German measles) similarly cause rash and fever illnesses that present clinically like measles infections. However, rubella infections generally lead to more mild clinical symptoms than measles infections for most individuals, with some rubella infections occurring asymptomatically. Unfortunately, when women experience a rubella infection during early pregnancy, the rubella virus can infect the fetus and cause a wide range of birth defects collectively referred to as congenital rubella syndrome (CRS) that can lead to lifelong disability [1,2].

Shortly after the introduction of measles vaccines in 1963, a March 1967 presidential statement launched the first US national measles elimination goal [3]. The first national measles elimination strategy notably included (i) the routine immunization of infants, (ii) the immunization of unvaccinated children entering school, (iii) improved disease surveillance, and (iv) the containment of outbreaks with immune globulin treatment and the immunization of susceptible individuals [4]. A 1963–1965 rubella epidemic led to highly visible and tragic cases of CRS that motivated intensive vaccine development efforts, which led to the introduction of rubella vaccines in 1969 [5]. Over time, two other national measles elimination initiatives followed in 1978 [6] and 1993 [7,8,9]. The US most likely stopped indigenous measles transmission by 1993 [10] and it convened an expert consultation that certified the accomplishment in 2000 [11]. After eliminating measles, the US turned its sights on rubella, and it successfully stopped indigenous rubella virus transmission by the early 2000s with the certification of the achievement in October 2004 [5].

Building on the success of the US and other countries, the entire western hemisphere (i.e., the Americas), led by the Pan American Health Organization (PAHO), set a goal in 1994 to eliminate regional indigenous measles virus transmission by the year 2000 [12], and established a goal in 2003 to eliminate indigenous rubella virus transmission by the year 2010 [13]. PAHO certified the accomplishment of the regional elimination of indigenous rubella transmission in 2015 [13] and indigenous measles transmission in 2016 [12]. These regional efforts helped to decrease the number of importations of both viruses into the US and reduced the risks of transmission restarting.

Despite these accomplishments, importations of measles and rubella viruses from other parts of the world continue(d) to pose a threat to Americans. The US continues to report measles cases associated with these importations, which lead to costly US measles outbreak response efforts [14,15,16,17,18,19]. In 2018, PAHO lost its regional measles elimination status due to the reestablished transmission of imported measles viruses in the Bolivarian Republic of Venezuela [20] and Brazil [21], and the US came close to losing its national measles elimination status due to a 2018–2019 outbreak in New York [22]. As of 2024, the national and regional elimination status of indigenous rubella transmission remains, although the US periodically reports clinical rubella and CRS cases associated with individuals infected outside the US.

### 1.1. US Measles and Rubella Vaccine and Immunization Policies

Measles and rubella immunization policies evolved over time. In 1963, the initial indication for the measles vaccine included healthy children 9 months and older and individuals in high-risk groups, who could receive one dose of a live attenuated measles vaccine or three doses of an inactivated measles vaccine given in monthly intervals, with the inactivated vaccine preferred only for individuals contraindicated for the live attenuated measles vaccine [23]. In 1965, the newly established Advisory Committee on Immunization Practice (ACIP) approved a routine immunization schedule that recommended the measles vaccine for children 12 months or older, due to interference between maternal antibodies and the live attenuated vaccine [24]. ACIP issued a 1965 statement in support of achieving high levels of immunization to achieve national measles elimination [25]. In 1966, ACIP changed its schedule to not recommend the inactivated measles vaccine, except for individuals contraindicated for the live attenuated measles vaccine [26]. In 1967, ACIP completely removed the inactivated measles vaccine from its list of recommended vaccines due to the evidence of atypical measles reported in children who received the inactivated measles vaccine and the limited effectiveness of the inactivated vaccine in immunocompromised children [27]. In 1969, ACIP recommended one dose of the rubella vaccine for children 12 months through puberty [28]. Following successful introduction and the prevention of rubella infections in children, ACIP extended its recommendation to include immunizing susceptible non-pregnant adolescent and adult women who agreed to prevent pregnancy for 3 months after receiving the vaccine, ideally with susceptibility to rubella demonstrated by serologic testing prior to immunization [29].

The separate administration of measles and rubella vaccines largely ended within a few years of rubella vaccine introduction. Following the licensure and demonstration of the non-inferiority of protection provided by combination vaccines containing both live attenuated measles and rubella antigens compared to the single antigen counterparts, ACIP recommended the use of the measles–rubella (MR) vaccine in 1971 [30]. This recommendation reduced the number of vaccine administrations and eliminated the previously recommended spacing of one month between doses of the single antigen live virus vaccines [30]. In the context of numerous outbreaks, ACIP made specific recommendations in 1971 for vaccinating or revaccinating children who lacked sufficient evidence of immunity, and encouraged restricting unvaccinated children from school attendance during outbreaks [31]. In 1976, ACIP recommended the measles–mumps–rubella (MMR) vaccine and further reconsidered the strategy of administration of more than one live virus vaccine antigen (e.g., MMR and the oral poliovirus vaccine) during a single clinical visit [32]. ACIP also changed the recommended age for receiving measles-containing vaccines (MCVs), MR and MMR at the time, to 15 months in 1976, with the further increase in age again due to interference with measles maternal antibodies [33]. In 1978, ACIP reconfirmed 15 months as the recommended age of receiving an MCV and its preference for the MMR formulation for childhood vaccination, and further emphasized the importance of vaccinating or revaccinating individuals lacking immunological protection from measles (including those who may have previously received the inactivated measles vaccine) [34].

The expansion of ACIP recommendations continued since the 1980. In 1982, ACIP added an international travel indication for measles vaccination for those born after 1956 without evidence of measles immunity [35]. In 1987, ACIP recommended that colleges require proof of measles vaccination for students prior to matriculation and that medical facilities require proof of measles vaccination for all employees born since 1956 and all employees with potential occupational exposure to measles virus [36]. Responding to the epidemiology of the time, in January 1989 ACIP issued a supplementary statement that recommended areas with recurrent measles outbreaks give infants a dose of a measles single antigen (i.e., monovalent measles) vaccine at 9 months of age in addition to the recommended MMR dose at 15 months [37]. In December 1989, ACIP changed its recommendation to a two-dose MMR schedule for all children: one dose at 15 months (or as early as 12 months in areas with recurrent outbreaks) and one dose at the age of school entry [38]. In 1998, ACIP changed the two-dose schedule to specify the delivery of the first MMR dose between 12 and 15 months and the second dose between 4 and 6 years of age, and it set a goal for all states to achieve the delivery of two MMR doses to all children prior to school entry by 2001 [39]. In 2005, the ACIP schedule recommended that children aged 12 months–12 years receive two doses of the MMR vaccine at least 1 month apart (with the recommended target ages for both doses unchanged from the 1998 recommendations [39]). The licensure of a combination vaccine that included measles–mumps–rubella–varicella (MMRV) in 2005 offered an alternative to separate MMR and varicella vaccines [40]. ACIP subsequently issued several related recommendations that did not impact the measles vaccine schedule or the choice of MMR or MMRV, henceforth MMR(V), and ACIP recommended administering the first doses at 12–47 months and second doses at 5–12 years of age [41,42,43,44]. As of 2024, the US routine immunization schedule continues to recommend two doses of MMR(V), with two vaccine formulations available: MMR (M-M-R-II™, North Wales, PA, USA and Priorix™, Philadelphia, PA, USA) and MMRV (ProQuad™, North Wales, PA, USA), with administration at ages of 12–15 months and 4–6 years and clearly indicated age ranges for catch-up [45].

### 1.2. Economic Analyses

Economic analyses of measles and rubella vaccines in the US performed over time emphasized the significant benefits of the vaccines, and many explored different immunization policy changes [46]. For measles, a 1969 economic analysis projected significant savings of lives and costs for the first 6 years of US measles immunization compared to no immunization with estimated net benefits of USD 423 million (USD 1962, which equals approximately USD 4.3 billion in USD 2023) [47]. A 1975 update strengthened the health and economic case for supporting measles immunization in the US during the first 10 years [48]. A 1978 economic analysis estimated a benefit–cost ratio of 10.3 and net benefits exceeding USD 1.1 billion (USD 1972, or USD 8.0 billion USD 2023) for the national measles elimination efforts between 1966 and 1974 [49]. Several economic analyses reported sustained significant net benefits of measles vaccination for the first 20 years of its use in the US [50,51,52], and characterized the savings associated with the shared administration of combination vaccines that included measles (i.e., MR and MMR) [51]. Consistent with the epidemiological importance of outbreaks around 1990, several economic analyses explored different options for outbreak response and the costs of outbreaks [53,54,55,56]. Around 2000, studies demonstrated significant potential savings for the US associated with the cessation of measles vaccination after global measles eradication [57], and emphasized the need to identify cost-effective strategies to increase measles immunization coverage [58].

Similarly, soon after the introduction of the rubella vaccine, economic analyses began exploring its health and financial benefits and the impacts of different US rubella immunization policies [46]. Based on an extrapolation of the costs of the 1963–65 rubella epidemic, an economic analysis of the US national rubella immunization policy of immunizing children 1–12 years old to avert the next expected rubella epidemic in the 1970s suggested that rubella immunization would need to cost more than USD 1 billion (USD 1964, USD 9.8 billion USD 2023) for the costs to exceed the benefits [59]. Two additional economic analyses estimated significant net benefits for the first 20 years of US rubella vaccine use [51,52]. An unpublished economic analysis in 1994 showed positive benefits of a two-dose MMR schedule, with the incremental costs of a second dose of MMR not exceeding the expected incremental benefits due to high first-dose coverage [60]. An analysis suggested that US rubella vaccination would provide benefits at least eight times greater than costs, with higher benefit–cost ratios associated with the use of MMR combination vaccines instead of the monovalent rubella vaccine and with various immunization policies and coverage assumptions [61].

Economic analyses of the current full childhood immunization schedule, including MRCVs, continue to show significant health and economic benefits compared to no vaccination [62,63,64] and the costs associated with importation-associated outbreaks [14,15,16,17,18,19]. Despite the numerous static economic analyses of the US investments in measles immunization [47,48,49,50,51,52,57,58,60,62,63,64] and rubella immunization [46,51,52,59,62,63,64] and many measles and rubella transmission models [65], no studies using a dynamic transmission model demonstrate the economic benefits of the more than five decades of the US use of measles and rubella vaccines. Prior studies demonstrated the importance of using dynamic models instead of static models to characterize the economics of interventions of communicable diseases theoretically [66,67] and for real diseases [68]. Guidelines for economic analyses recommend the use of dynamic transmission models for economic analyses for vaccine-preventable diseases [69,70,71]. This analysis fills the gap of using a dynamic transmission model to characterize the health and economic benefits of the US measles and rubella immunization programs.

## 2. Materials and Methods

To characterize the health and economic outcomes of the actual US program, this analysis considered the reported measles and rubella cases and estimates of cases using an existing dynamic disease transmission model for measles and rubella [72]. Briefly, the model tracked the births, deaths, aging, and immunity of all individuals in the US population as it evolves through the following immunity states: maternally immune (i.e., infection-induced and vaccine-induced tracked separately), susceptible, exposed, infected (and infectious), recovered, or vaccinated. The model also tracked the progression of pregnancies to simulate the impacts of infections on pregnancy outcomes and cases of CRS [72]. This analysis updated the time-related model inputs for epidemiology, demographics, immunization, and costs through the end of 2023. The model time horizon extended from 1963 to 2030, and assumed the prospective use of two doses of MMR(V) for 2024–2030. The analysis used standard methods to characterize the societal health and financial costs, including the use of a discount rate of 3% and the computation of incremental cost-effectiveness ratios (ICERs) as well as incremental net benefits (INBs). The model estimates provided comparisons between the actual historical US immunization program use of MRCVs (i.e., including all of the changes that occurred over time in schedules and formulation changes) and a counterfactual world with no measles or rubella vaccine use.

### 2.1. Transmission Model Input Updates

**Figure 1:**
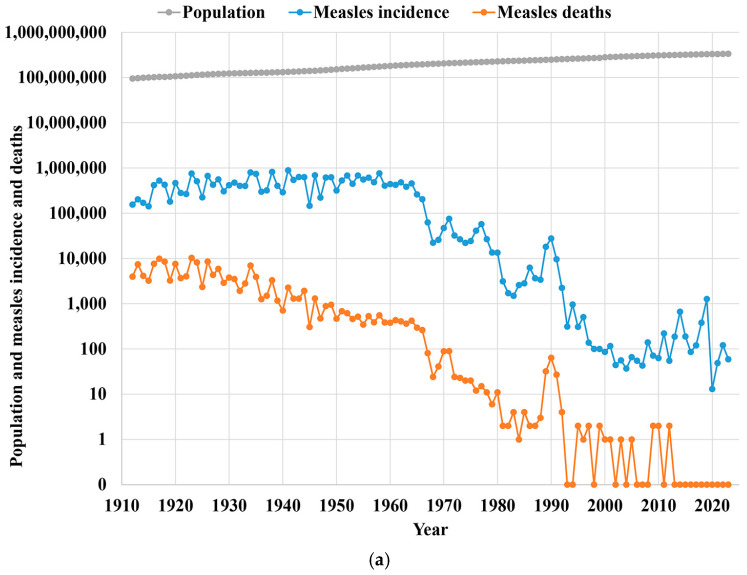

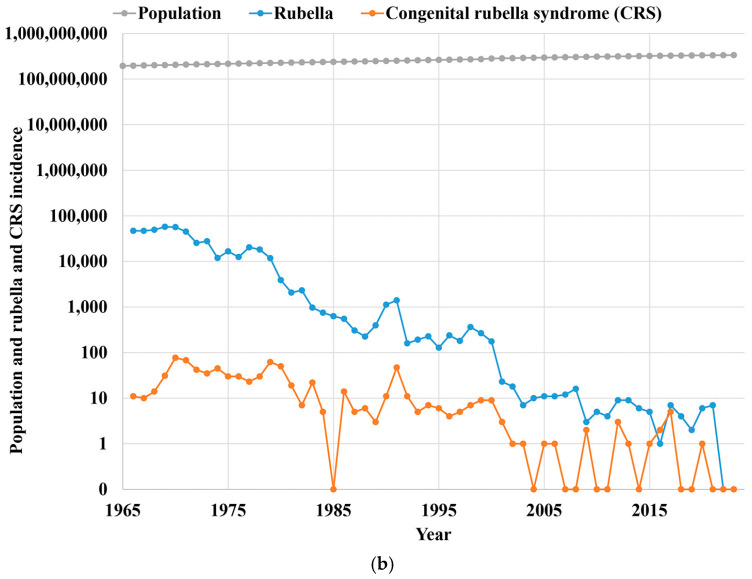
Trends for US population growth [74,75,76] and (**a**) reported measles incidence [73,77,78,79,80,81] and deaths [78,79,82] between 1912 and 2023 and (**b**) reported rubella incidence and congenital rubella syndrome (CRS) cases [73,77,80,82] between 1965 and 2023.

Measles became a notifiable disease in the United States in 1912, with 19 states reporting initially, all states reporting by 1928, and the US Centers for Disease Control and Prevention (CDC) taking on the responsibility for data collection and synthesis starting in 1961 [73]. Due to their similar clinical presentation, prior to the 1960s, reported measles cases probably included some rubella cases. Rubella became a reportable disease in 1966 with the widespread utilization of laboratory testing facilitating its distinction from other fever and rash diseases [73]. Figure 1 shows the steady and substantial increase in the estimated size of the US population [74,75,76] for (a) 1912–2023 along with reported measles cases [73,77,78,79,80,81] and deaths [78,79,82], and (b) 1966–2023 along with reported rubella and CRS cases [73,77,80,82]. While the US population increased by a factor of 3, Figure 1a shows the rate of measles incidence and deaths varied significantly prior to the introduction of the measles vaccine in 1963, and then dropped significantly following its introduction. These data imply a substantial reduction in the reported measles case fatality rate over time, which most likely reflects improvements in health care (e.g., detection, treatment, case management, and overall health systems). Similarly, rates of rubella and CRS incidence declined significantly over time following the introduction of the rubella vaccine in 1966 (Figure 1b).

The quality of measles surveillance in the US changed significantly over time. Estimates suggest that in the pre-vaccine era through the early 1970s, reporting most likely captured less than 10% of all cases [83,84,85]. A review of the completeness of measles reporting during the 1980s and 1990s suggested a wide range of 3–58% [86]. Given the substantial underreporting, the early measles incidence data (Figure 1a) most likely provide some indication of the years of major outbreaks (i.e., the peaks and troughs of cases), but not the absolute numbers of cases. However, some uncertainty related to the peak years comes from the potential impact of major rubella epidemics contributing to measles case counts due to the reliance on syndromic surveillance [72]. In contrast to measles, given the relatively short period of time between rubella becoming notifiable and the introduction of the rubella vaccine, relatively little information exists to assess the extent of the underreporting of rubella infections prior to vaccine introduction. However, given the substantial underreporting for measles, the use of syndromic surveillance for both diseases, and the relatively less severe clinical presentation of rubella, substantial underreporting most likely occurred for rubella and CRS. As the US pursued and achieved the elimination of indigenous measles and rubella transmission, surveillance continued to improve (as required to confirm the achievement of the goals), but some underreporting of cases still occurs. Nonetheless, improvements in laboratory methods allowed the US to document the end of the transmission of indigenous rubella lineages [87] and measles lineages and identify the likely sources of imported measles and rubella viruses after the national elimination of indigenous transmission [10,88]. Given the uncertainty about the timing, location, and transmission of future importations of measles and rubella into the US, for 2024–2030 this analysis prospectively assumed 250 annual measles cases, 4 annual rubella cases, and 1 annual CRS case. These assumptions may over- or under-estimate the cases that will occur, because the actual size of outbreaks depends on the timing of introduction and the level of under-immunization in the outbreak communities. For example, as shown in Figure 1a, relatively larger recent measles outbreaks occurred during the winters of 2014–2015 and 2018–2019, and cases dropped substantially in 2020 due to reductions in social mixing and travel during the COVID-19 pandemic.

This analysis started with estimates of historical routine immunization coverage for MCVs (i.e., monovalent measles, MR, MMR, and MMRV) and rubella-containing vaccines (RCVs, i.e., monovalent rubella, MR, MMR, and MMRV) in the US based on prior work [72,89]. A closer review of the use of measles and rubella vaccines in the US and the evolution of ACIP policies (described above) led to some additional considerations for the model. In 1963, the US introduced two types of licensed injectable measles vaccines: inactivated and live attenuated. The inactivated vaccine represented about one-third of total doses used in 1963 and required 3 doses per child, which implied a higher cost per fully immunized child. The model accounted for this requirement of multiple doses for the inactivated vaccine in the coverage estimates for 1963–1966. Many recipients (approximately 50%) of the initial live attenuated measles vaccine developed symptoms of fever and rash, which motivated the development of a further attenuated live measles vaccine with significantly lower side effects [90]. Similarly, the initial rubella vaccines introduced varied with respect to their immunogenicity and reactivity, but by 1979 the RA27/3 rubella vaccine emerged as the national preference [91].

**Figure 2:**
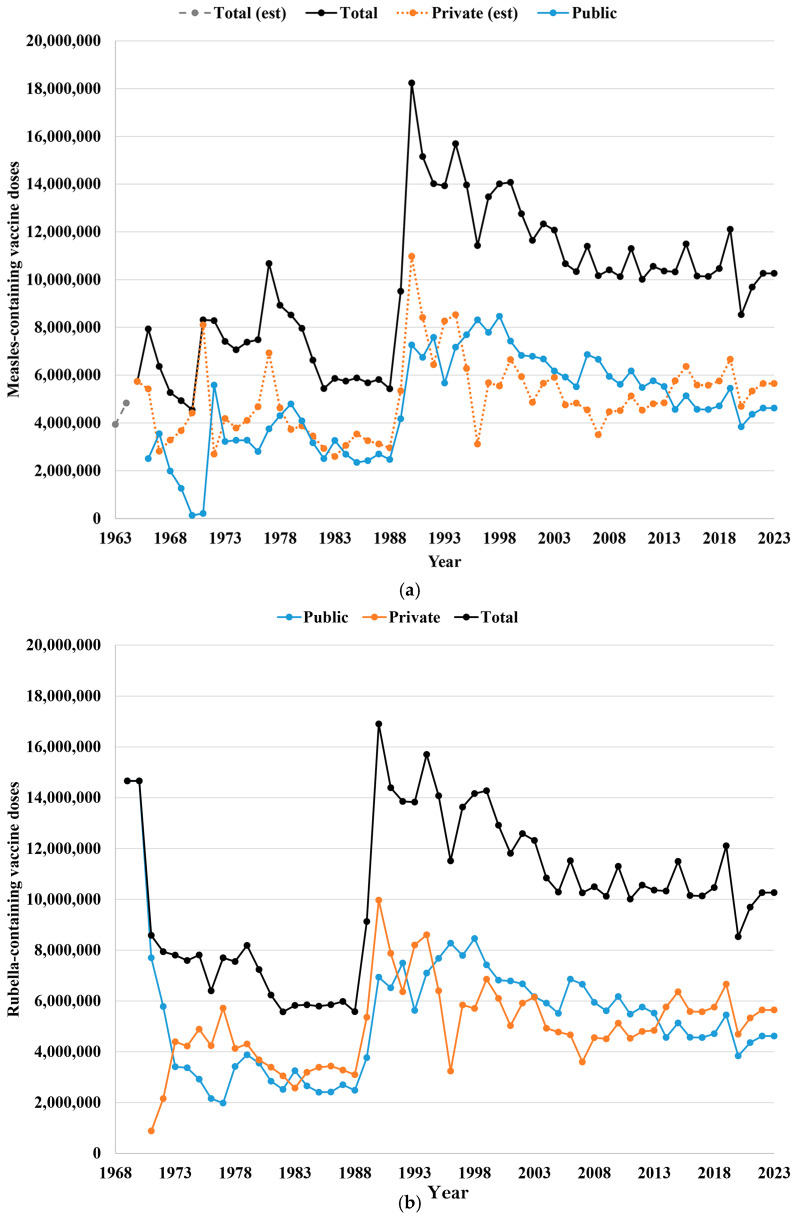
Number of vaccine doses by payer type and in total in the United States for (**a**) measles-containing vaccine doses since 1963 and (**b**) rubella-containing vaccine doses since 1969 with dashed lines showing estimates (see acknowledgments at the end). Total measles doses for 1963 and 1964 estimated [48]; reporting of total measles doses started in 1965; reporting of public doses started in 1966; reporting of total rubella doses started in 1969; and public doses estimated as 45% of total for 2016–2022.

In the US, individuals receive health care (including immunizations) paid for by public sources (i.e., the government) or private sources (e.g., self-pay or employer-based health insurance). Figure 2a shows the estimated number of MCV doses purchased by year in the US since 1963 by payer type and in total, and Figure 2b shows the estimated number of RCV doses purchased by year in the US since 1969 by payer type and in total. For the rubella dose estimates in 1969 and 1970, this analysis apportioned the estimated total rubella vaccine doses purchased by 1970 as equivalent for 1969 and 1970 and assumed 90% public purchase and 10% private purchase. The CDC began purchasing MCV doses following the approval of federal funds for 1966–1968 [92] to support national measles elimination in 1967 [4]. After the licensure of the rubella vaccine in 1969, Congress allocated federal funds for RCV purchase, instead of for measles immunization, between 1969 and 1971, since it prioritized the introduction of the rubella vaccine [92]. Following a resurgence of measles incidence in 1969–1971, federal funds began to support the public purchase of measles and rubella vaccines individually and then in the combination MR formulations. The purchase of MRCVs increased as school immunization requirements started to come into effect in some states in the early 1970s.

In 1989 and 1990, vaccine demand increased consistent with the shift to a two-dose immunization schedule. The demand increase included just over 1 million monovalent live measles vaccine doses, which aimed to increase measles protection in adolescents and young adults who received the inactivated measles vaccine as children. In addition, the US military delivered one dose of MCV to all military recruits (i.e., approximately 200,000 doses per year) between 1980 [93] and the mid-2000s [94], after which time the military services began screening recruits and selectively vaccinating only those lacking immunity. Based on data prior to 2015, this analysis assumed a public purchase of 45% of total doses for 2016–2024. Although the fraction of all MRCV doses in Figure 2 given to Americans of different ages remains uncertain, consistent with immunization recommendations for travel, military personnel, unvaccinated women of childbearing age, and health care providers, this analysis assumed that 5–10% of the annual MRCV doses have gone to individuals over 17 years old after 1980.

**Figure 3:**
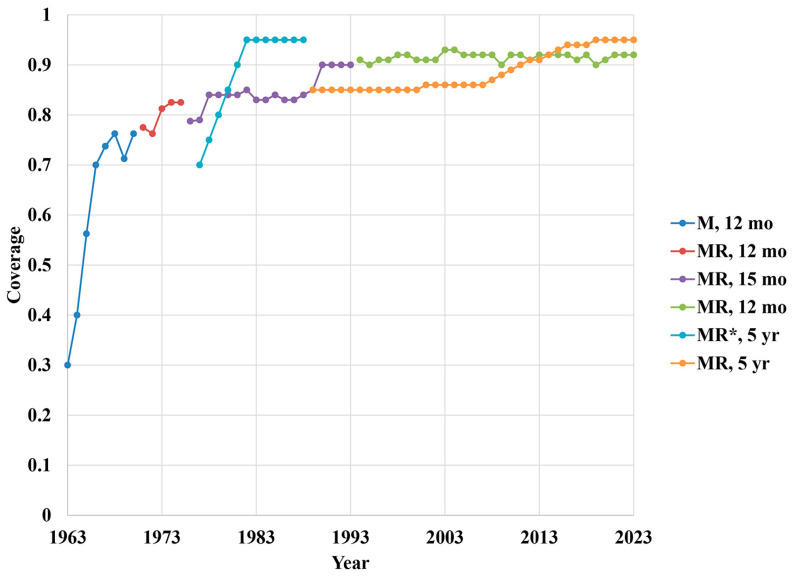
Assumptions about routine immunization coverage of measles- and rubella-containing vaccines by year and age of administration. * Second opportunity for unvaccinated children aged 5 years to receive the measles vaccine, to account for the increasing adoption of school immunization requirements (not a second dose).

Figure 3 summarizes (a) MCV and (b) RCV routine immunization coverage estimates by year by the age of vaccine delivery [89]. For 2023 on, this analysis assumed the same routine immunization schedule and coverage rates as occurred in 2022 and that the other vaccine doses purchased include supplemental immunization activities, including campaigns performed to introduce both vaccines, outbreak response, and the catch-up of older individuals (e.g., military and women of childbearing age) as well as wastage. For 1977 to 1988, this analysis included a second opportunity for unvaccinated children aged 5 years to receive the vaccine, to account for the increasing adoption of school immunization requirements (see * in Figure 3). The administration costs of MCVs and RCVs depended on the number of antigens in the vaccine formulation, because the multiple antigens share the costs for the single injection.

**Figure 4:**
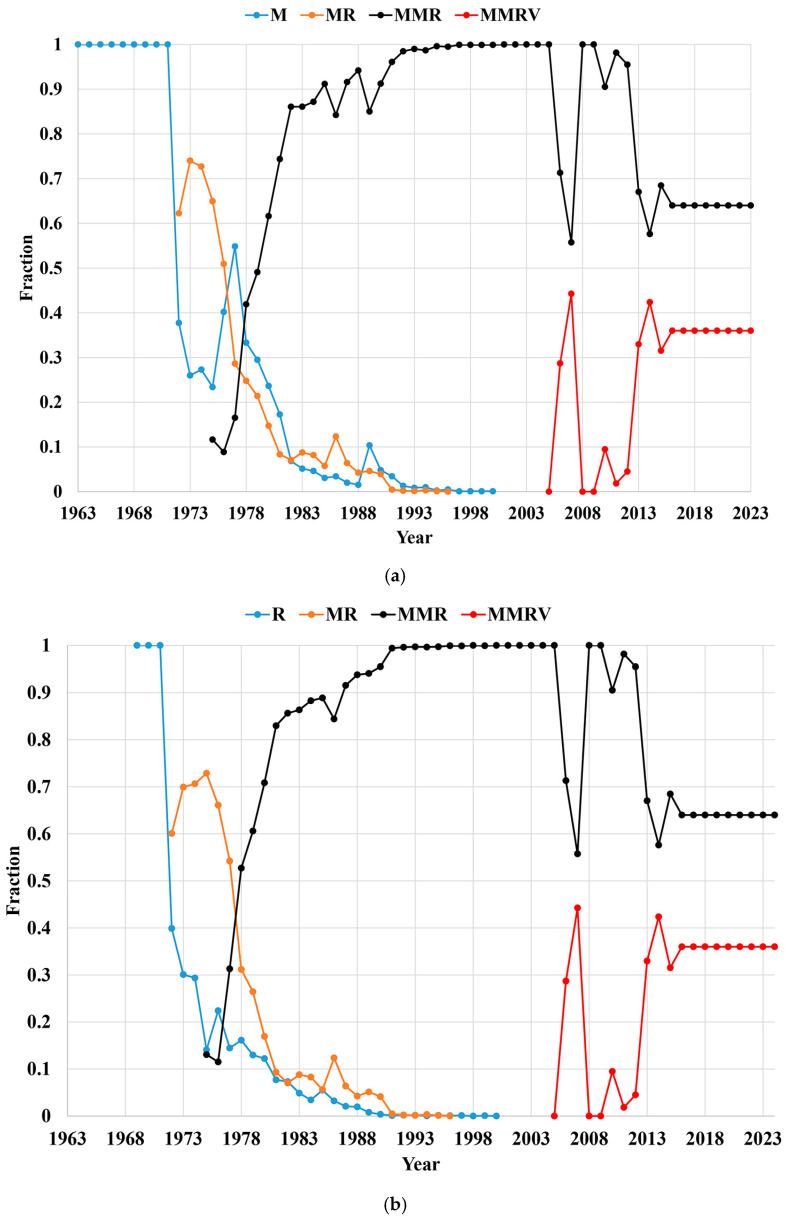
Fractions of the different (**a**) measles- and (**b**) rubella-containing vaccine formulations used in the public sector by year since 1963.

Figure 4 shows the fractions of the different MRCV formulations used for the public sector doses by year. Slow uptake and supply disruptions of MMRV explain some of the variation in the fractions of MMR and MMRV in Figure 4. For 2016 on, this analysis assumed the public delivery of 65% of all MMR doses and 35% of all MMRV doses.

### 2.2. Cost Input Updates

**Figure 5:**
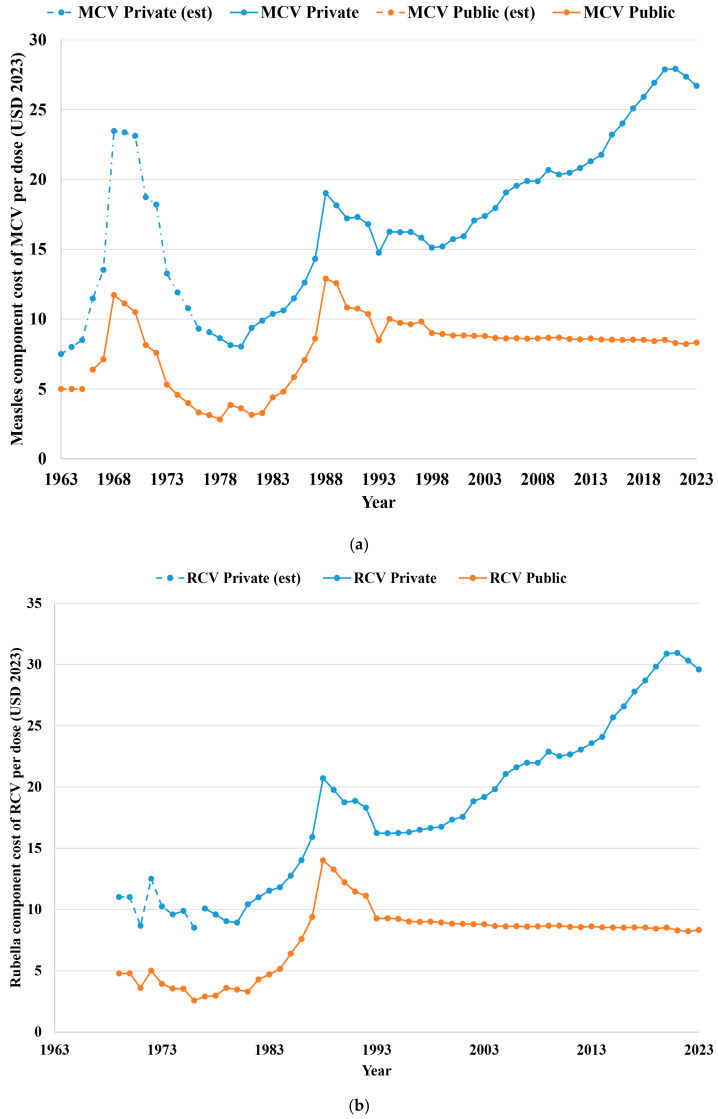
Estimated cost per dose of the (**a**) measles component and (**b**) rubella component by public and private purchase.

For consistency, the analysis reported all costs in 2023 US dollars (noted USD 2023) adjusted using the consumer price index [95]. Figure 5 summarizes the (a) measles component price for MCVs and (b) rubella component price for RCVs for the public and private sectors (converted to USD 2023) based on data obtained from the published literature [96] and unpublished data provided to the CDC by vaccine manufacturers. The cost estimates accounted for the estimated numbers of doses for each vaccine presentation (Figure 2 and Figure 4), the applicable federal excise taxes, and the estimated cost of the specific antigen component in the combination vaccines. In 1987, vaccine purchases (both public and private) became subject to a federal excise tax [97] used to create a fund to support the Vaccine Injury Compensation Program [98]. A federal excise tax of USD 4.44 per dose (nominal) applied for MMR for 1987–1992 [97]. On August 10, 1993, the federal excise tax became permanent [99] and the amount for MMR of USD 4.44 per dose (nominal) resumed. In August 5, 1997, the Taxpayer Relief Act of 1997 [100] lowered the federal excise tax to USD 0.75 per antigen (nominal, i.e., USD 2.25 per MMR dose), and the tax continues at that amount to date. This analysis assumed that the nominal federal excise tax will continue to be USD 0.75 per antigen in perpetuity, which implies a gradual decline in real cost over time. The drop in the cost in 1993 in Figure 5 reflects the absence of a federal excise tax for part of that year (the analysis assumed the tax applied to 50% of the MRCV vaccine supply in 1993, which includes tax applied to the 40% of annual doses sold after 9 August 1993 and 10% more to account for some of the floor tax imposed for doses held on 10 August 1993). To obtain the cost of the measles component in the mixture of all MCVs each year, the analysis assumed that the measles component accounts for 60% of the antigen cost of MR and 30% of the antigen cost of MMR, and the same cost for the measles component in MMR and MMRV for both the public and private sector costs. The model assumed that the rubella component accounts for 40% of the antigen cost of MR, 30% of the antigen cost of MMR, and the same cost for the rubella component in MMR and MMRV for both the public and private sectors. The Vaccines for Children program [99] mandates that the contract price for public purchases of some vaccines (including MRCVs) cannot increase more than the pre-excise tax public price of the vaccine in 1 May 1993 adjusted using the consumer price index [95], which explains the slightly decreasing public real cost of MRCVs (in USD 2023) in Figure 5 after 1993. The analysis assumed a constant public cost for the measles and rubella components of MRCVs from 2023 through the rest of the analytical time horizon. In contrast, consistent with the trend in Figure 5, the model assumed that the private cost will increase at a rate of USD 0.36 per year from 2023 to 2030 (based on a linear regression of historical private cost increases, R^2^ = 0.96).

This analysis based its non-vaccine cost and valuation estimates on the values for high-income countries reported previously [1]. Table 1 summarizes the updated or new estimates for non-vaccine costs per injection (including administration, needle disposal, etc.) and other costs per measles or rubella infection or per dose for MRCVs, all appropriately updated to USD 2023. The baseline analysis did not include costs related to measles or rubella research, surveillance, or program management related to achieving the national control and elimination goals (i.e., programmatic costs).

## 3. Results

**Figure 6:**
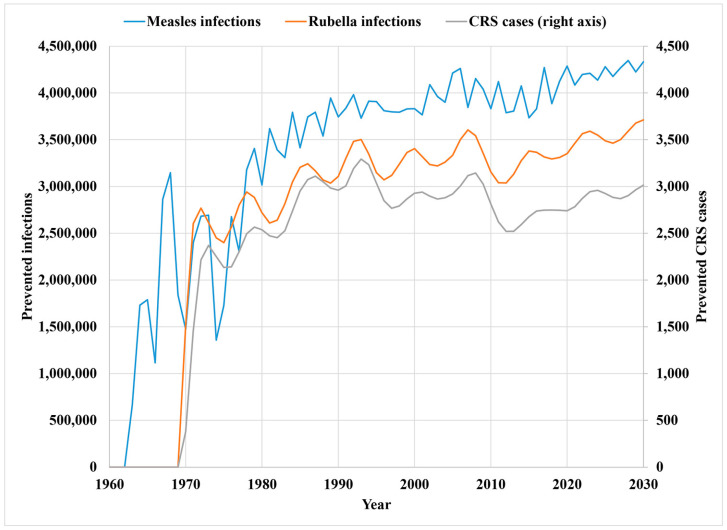
Estimates of prevented annual measles and rubella cases (left axis) and CRS cases (right axis) in the United States for 1963–2030 based on differences between the modeled US immunization program and the counterfactual scenario of no immunization.

Figure 6 shows the model estimates of prevented cases of measles and rubella (left axis) and CRS (right axis) by the US immunization program (compared to no measles or rubella vaccines). The results show the increase in prevented cases following the introduction of the measles vaccine in 1963 and rubella vaccine in 1969, with the measles cases prevented approaching the size of the annual US birth cohort. A comparison of the prevented rubella (left axis) and CRS (right axis) cases shows a slight shift to the right for the CRS cases, which corresponds with the time between early trimester infections and CRS diagnosis after live births. In Figure 6, after the national elimination of the transmission of indigenous viruses, this analysis subtracts the reported cases from the estimated cases from the modeled counterfactual with no immunization up to and through 2023 and assuming average imported cases for 2024–2030 (see Section 2.1).

Table 1 summarizes the results of the economic analyses. For the program of actual US investments, Table 1 shows total costs for US investments in measles immunization for 1963–2023 of USD 8.1 billion and in rubella immunization for 1969–2023 of USD 14.1 billion. The analysis estimates an additional approximately USD 1.2 billion for measles immunization and USD 1.5 billion for rubella immunization expected for 2024–2030. Historical and future US investments prevented an estimated approximately 237 million measles infections, 228,000 measles deaths, 193 million rubella infections, and 166,000 CRS cases. These investments imply net benefits (from avoided treatment costs minus immunization costs) of approximately USD 310 billion for measles and USD 430 billion for rubella and CRS, even without incorporating avoided productivity losses and intangible costs. Including productivity savings estimated by valuing each DALY lost as equivalent to per capita gross national income [1], this analysis estimates incremental net benefits of immunization in the US compared to no immunization of over USD 1.2 trillion for measles for 1963 to 2023 (with USD 200 billion additionally anticipated for 2024–2030) and over USD 880 billion for rubella for 1969 to 2023 (with USD 160 billion additionally anticipated for 2024–2030). Due to importations, the US continues to incur some financial costs associated with investigations by public health authorities, particularly for measles outbreaks. Since 1980, this analysis estimates that cumulative outbreak response costs most likely exceeded USD 30 million. US investments in measles and rubella immunization continue to provide enormous savings of human and financial costs, and global eradication of both diseases would offer some additional savings due to avoided outbreaks, if achievable.

## 4. Discussion

US investments in measles and rubella immunization continue to provide enormous net savings of human and financial costs for Americans. While these results apply to the US, the wealth of economic analyses related to measles immunization imply similar expectations of significant savings for other countries, regions, and globally [46]. A 2016 analysis that characterized the benefits of vaccines for 94 low- and middle-income countries for 2011–2020 reported approximately USD 58 (USD 2010) returned per dollar spent on measles vaccination, and showed the measles vaccine as the most net beneficial vaccine delivered among the 10 commonly delivered childhood vaccines considered in the analysis [101]. A recent analysis identified reductions in measles mortality as responsible for over 80% of the global reduction in mortality by all vaccine-preventable diseases during the first 50 years of the Expanded Program on Immunization (EPI) [102].

Despite regional measles and rubella elimination efforts, importations of measles and rubella viruses into the US remain an ongoing threat due to the possibility of any unvaccinated American coming into contact with an infected individual (e.g., while traveling in measles- and/or rubella-endemic countries or with an infected individual traveling within the US). Recent outbreaks demonstrate the potential for restarting transmission in under-vaccinated communities. By extension, this and other analyses suggest that the global eradication of measles and rubella virus transmission could lead to significant savings for the US and globally, and this continues to motivate national investments in the global immunization and potential eradication of measles and rubella. However, the US appears unlikely to ever stop its immunization with MRCVs, and consequently, recognizing an eradication dividend largely depends on potential savings of avoided treatment costs and national outbreak response.

Although relatively minor with respect to both health and economic impacts, this analysis includes the quantification of very rare, but still non-zero, adverse vaccine-associated events. In theory, with global eradication (i.e., no more measles or rubella transmission or infections), the costs of these rare events could exceed the consequences of infections in the US, which might lead to discussions about ending immunization. However, as of 2024, global concerns about biological agents remain heightened by the recent emergence of the SARS-CoV-2 virus that led to the global COVID-19 pandemic. Ending immunization for measles and/or rubella in the US would not likely find support with US public health authorities.

All models come with limitations. Given the limited information and the need to reconstruct historical information for retrospective model inputs and extrapolate for future model inputs, the model results inherently include uncertainty about how well the model estimates represent the past and/or will predict the future. The model includes numerous assumptions that simulate transmission artificially (e.g., assuming more homogeneity in the population than exists), but produces estimates consistent with the available evidence. In addition, the results of any economic analysis depend on the assumptions made related to valuation over time (e.g., discounting future costs and benefits). The major insight of very significant benefits remains highly robust to changes in the assumptions: measles and rubella immunization lengthens and improves the quality of lives and saves money. Thus, this analysis emphasizes the overwhelming results of substantial health and economic savings from measles and rubella vaccine use in the US, while noting some uncertainty about the absolute numbers and the lack of inclusion of all costs (e.g., unquantified non-vaccine-related programmatic costs).

Combining this analysis with a similar analysis for polio [68] and prospective analyses of the full national childhood immunization program, the US has benefitted and continues to benefit very significantly from its investments in childhood immunization programs. Given the ability of measles and rubella vaccines to prevent bad and costly outcomes, Americans do not experience the events that do not happen. While fortunately enjoying the benefits of prevention, this unfortunately leads to some lack of appreciation of the important health and economic benefits of these vaccines. Studies like this should help provide some insights about what might occur in the absence of vaccines. Those who remember and/or understand the tragic outcomes caused by vaccine-preventable diseases will need to continue to help those with no or little experience with the diseases to fully appreciate the value of vaccines. Measles and rubella importations remain a very real threat, and all individuals remain vulnerable to the potential adverse health outcomes associated with measles and rubella infections so long as they remain unprotected.

## 5. Conclusions

US investments in measles and rubella immunization continue to provide enormous savings of human and financial costs and to prevent substantial mortality and morbidity.

## Figures and Tables

**Table 1:** Costs and effectiveness of measles immunization programs in the United States (USD 2023).

**Metric**	**Measles**		**Rubella**	
Introduction *–2023	No vaccine	Vaccine	No vaccine	Vaccine
Infections (millions)	228	20	172	5
Measles deaths (thousands)	222	20		
CRS cases (thousands)			152	7
Treatment costs (billions)	307	26	400	13
DALYs lost (millions)	17	1	8	0.2
Vaccine doses (millions)		583		546
Immunization costs (billions)		8		14
Productivity losses (billions)	985	55	516	10
2024–2030				
Infections (millions)	30	0.002	25	0.00003
Measles deaths (thousands)	26	0.003		
CRS cases (thousands)			21	0.007
Treatment costs (billions)	38	0.003	59	0.009
DALYs lost (millions)	2	0.00009	1	0.0001
Vaccine doses (millions)		72		56
Immunization costs (billions)		1		2
Productivity losses (billions)	167	0.007	104	0.01
**Economic analysis summary**	**Measles**	**Rubella**	**Total**	
Introduction *–2030				
Incremental immunization costs (billions)	9	16	25	
Incremental treatment cost (billions)	−320	−446	−765	
Incremental cost–effectiveness ratio	CLS	CLS	CLS	
Incremental net benefits (INBs) (billions)	310	430	740	
Productivity costs saved (billions)	1097	610	1706	
INB with productivity costs (billions)	1407	1040	2447	

Abbreviations: CRS, congenital rubella syndrome; DALY, disability adjusted life years; INB, incremental net benefits. * Introduction year 1963 for measles vaccine and 1969 for rubella vaccine.

## Data Availability

No new data were created or analyzed in this study. Data sharing is not applicable to this article.
